# Dental fluorosis in populations from Chiang Mai, Thailand with different fluoride exposures - Paper 2: The ability of fluorescence imaging to detect differences in fluorosis prevalence and severity for different fluoride intakes from water

**DOI:** 10.1186/1472-6831-12-33

**Published:** 2012-08-21

**Authors:** Michael G McGrady, Roger P Ellwood, Patcharawan Srisilapanan, Narumanas Korwanich, Andrew Taylor, Michaela Goodwin, Iain A Pretty

**Affiliations:** 1 School of Dentistry, University of Manchester, Manchester, M13 9PL, England; 2 Colgate Palmolive Dental Health Unit, 3A Skelton House, Lloyd Street North, Manchester, M15 6SH, England; 3 Faculty of Dentistry, Chiang Mai University, Chiang Mai, Thailand

## Abstract

**Background:**

To assess the ability of fluorescence imaging to detect a dose response relationship between fluorosis severity and different levels of fluoride in water supplies compared to remote photographic scoring in selected populations participating in an observational, epidemiological survey in Chiang Mai, Thailand.

**Methods:**

Subjects were male and female lifetime residents aged 8-13 years. For each child the fluoride content of cooking water samples (CWS) was assessed to create categorical intervals of water fluoride concentration. Fluorescence images were taken of the maxillary central incisors and analyzed for dental fluorosis using two different software techniques. Output metrics for the fluorescence imaging techniques were compared to TF scores from blinded photographic scores obtained from the survey.

**Results:**

Data from 553 subjects were available. Both software analysis techniques demonstrated significant correlations with the photographic scores. The metrics for area effected by fluorosis and the overall fluorescence loss had the strongest association with the photographic TF score (Spearman’s rho 0.664 and 0.652 respectively). Both software techniques performed well for comparison of repeat fluorescence images with ICC values of 0.95 and 0.85 respectively.

**Conclusions:**

This study supports the potential use of fluorescence imaging for the objective quantification of dental fluorosis. Fluorescence imaging was able to discriminate between populations with different fluoride exposures on a comparable level to remote photographic scoring with acceptable levels of repeatability.

## Background

The measurement of the prevalence and severity of enamel fluorosis in populations for both epidemiological purposes and the evaluation of fluorosis risk associated with therapeutic interventions has traditionally been carried out using clinical indices such as Dean’s Index [1], the Fluorosis Risk Index (FRI) [2], Thylstrup and Fejerskov Index (TF) [3] and the Tooth Surface Index of Fluorosis (TSIF) [4]. The use of each of these indices requires an examiner to visually assess a tooth surface and by using predetermined criteria allocate a score as an interpretation of the aetiology and severity of the clinical presentation. Despite the wealth of historical data from studies using clinical indices criticism of their use exists [5-8]. This is particularly true when considering the fact the indices are subjective and can be prone to bias (knowledge of the fluoridation status of a population under examination), inter-examiner differences and personal thresholding associated with the presentation of fluorosis at low levels of severity [9,10]. This results in difficulties during the comparison and interpretation of multiple studies that have used subjective indices. It is possible to avoid the “blinding” issue of clinical examinations by moving the population to a central or distant location for examination, but this can be associated with logistical issues [11,12]. Remote scoring of clinical photographs can address issues of blinding so examiners have no knowledge of the fluoride exposure of the subjects under assessment [10]. This method of assessment can provide data considered to be more robust when compared to data obtained from direct clinical assessment. There are additional benefits with the use of clinical photographs. It is possible to capture digital images that are not only of high quality but can be archived and used for longitudinal and repeat assessments, clinical and research governance and audit processes.

Although the scoring of clinical photographs may address potential bias from blinding and carries advantages over direct clinical assessment, it still relies upon the application of a subjective index by an examiner that is still prone to such issues as personal thresholding and variability between and within examiners. In addition, the magnification of images could result in a tendency to over score fluorosis for milder severities.

Alternative means of assessing fluorosis by methods that are both quantitative and objective would be considered desirable. The possibilities of optical techniques with and without the diagnostic judgment of a clinician have been explored [5]. The ability to quantify demineralization in early enamel lesions has been demonstrated and validated using changes in fluorescence [13]. The technique quantifies the loss of fluorescence due to demineralization of enamel in a lesion relative to the surrounding sound enamel providing information on the percentage fluorescence loss (ΔF) relative to sound surrounding enamel and the Area (mm^2^) in which this loss of fluorescence occurs. The determination of overall mineral loss (ΔQ) is a metric derived from the product of ΔF and Area.

As both dental caries and enamel fluorosis are phenomena relating to hypomineralized enamel, an opportunity to objectively quantify fluorosis arises. Confounding factors exist that complicate this approach. Fluorescence imaging relies upon the image analysis software to reconstruct the lesion relative to sound surrounding enamel i.e. mineral loss occurring as discrete lesions. Fluorosis differs in its appearance as it presents as diffuse lesions that may extend across the whole tooth surface [14]. This prevents the use of image processing techniques used in the assessment of carious lesions being employed to quantify fluorosis as it becomes more difficult to reconstruct defuse lesions relative to sound enamel.

Novel software techniques and imaging systems have been developed in order to utilize fluorescence imaging in order to assess and objectively quantify enamel fluorosis and these have been tested in vivo [15]. It was found it was possible to quantify fluorosis using fluorescence imaging and overcome the issues associated with the assessment of diffuse lesions with no clear sound area to act as reference. Using this technique an image blurring methodology was applied to the green channel of the bitmap image obtained from fluorescence imaging. The blur technique involved the averaging of pixels within a matrix of pre-determined size replacing each point in the image with the average value of the surrounding pixels. The greater the size of the matrix, the larger the blur effect as more pixels are averaged. On completion of the blur process the “unsharp-mask” was subtracted from the original image leaving those areas considered to be fluorosis. The blur image acts as the control or sound area required for reconstruction of the lesion. The authors decided the optimum parameters were found by employing a blur effect at 30 pixels with a pixel selection of 2 standard deviations from the base level. This had the highest correlation with the clinical scores using TF index (Kendall’s Tau 0.869) when the metric of ΔQ_blur_ was chosen as the summary variable. Artifacts created by the blur technique tended to underestimate both the fluorescence loss (ΔF_blur_) and Area_blur_, particularly at higher levels of fluorosis severity where there is less “sound” enamel to act as a reference.

The purpose of this study was to further develop the use of fluorescence imaging for the analysis of fluorosis. The study aimed to examine a population with a wide range of fluoride ingestion from drinking and cooking waters and hence potential fluorosis experience. This approach provides a wide range of fluorosis presentation to assess the system’s ability to detect a dose response to changes in fluoride exposure from water sources when compared to a randomized blinded score of TF index obtained from conventional digital photographs. The study also aimed to evaluate the use of an alternative system of analysis for the fluorescence images in order to address the issues relating to the artifacts created with the existing blur technique and the resulting effects on the metrics of ΔF_blur_ and Area_blur_.

## Methods

### Screening and selection of subjects

Subjects selected for this study had participated in an epidemiological survey looking at fluorosis in Chiang Mai, Thailand. The protocol for the study was approved by Human Experimentation Committee, Faculty of Dentistry, Chiang Mai University, Thailand (clearance number 1/2008) (with notification to the University of Manchester Committee on Ethics on Research on Human Beings). The subjects were healthy males and females aged 8-13 years old. Written consent was obtained from the subjects and their parents. Water samples were collected from all consented subjects in order to determine fluoride content for both the drinking water supply and the cooking water supply. Where a common water supply was used, a single sample analysis was undertaken. Water sample analysis was carried out according to an analytical protocol by the Science and Technology Service Centre, Chiang Mai University. The fluoride content of the samples was determined using a 4-Star Benchtop pH/ISE meter, Orion Company, Mass, USA. The subjects were assigned to groups of different water fluoride content intervals based upon the data generated from the cooking water samples. This was owing to the fact there was a wider range and variation in the fluoride content of the cooking water compared to the drinking water. The aim was to recruit equal numbers of subjects into groups representing a range of fluoride concentration in the water supply.

**Figure 1:**
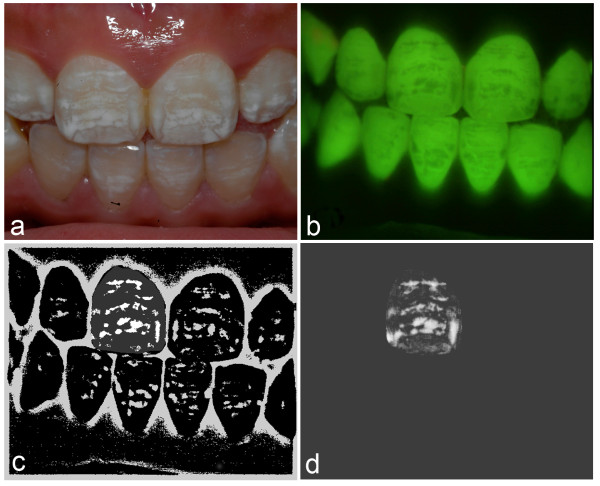
**Images demonstrating fluorosis analysis. a**. Conventional digital image of a subject presenting with fluorosis. **b**. Fluorescence image captured demonstrating fluorosis (areas of florescence loss). **c**. Output from analysis using existing technique. **d**. Output from analysis using convex hull technique. (Image adjusted for contrast for illustrative purposes).

Consented subjects were recruited on the basis of the fluoride content of drinking and cooking water samples and were assigned a five-digit subject ID number. The first two digits specified the school and the next 3 digits the subject’s individual study number based on the sequence of their recruitment. During the observational survey all subjects had standardized conventional digital photographs taken of the maxillary central incisors after the teeth had been cleaned and dried [16]. An example image is illustrated in Figure 1a. A consensus score by two examiners (RPE, MGM) based at a remote location was performed on the images that were presented in a randomized and blind manner.

### Fluorescence image capture

The imaging equipment comprised a high-resolution 3 CCD camera (Jai M91P, Jai Corp., Copenhagen, Denmark) fitted with a 16-mm F1.4 lens (Pentax, Slough, UK) and a long-pass yellow filter (495 nm, Schott, Stafford, UK). The light source was a custom made LED array with variable illumination emitting light with peak source at 405-nm. A custom-built stabilizing unit, comprising an adjustable head and chin support and a camera focus platform to which the camera and illuminator were connected enabled the camera to be moved and focussed while the subject remained static Figure 1b).

A number of subjects were randomly invited to have repeat fluorescence images taken in order to assess the repeatability of the image capture and image analysis procedures.

### Software

The software used for the existing technique utilized MATLAB version 7.6.0 (R2008a, Mathworks, N.Y., USA) image processing software to analyze the bitmap images obtained from the fluorescence image capture. A series of process applications included the image blur, the subtraction mask and the analysis of the resultant image (Figure 1c). The technique is described in detail in the literature [15].

An alternative analysis software was utilized that was originally designed to quantify stain on teeth [17]. The hypothesis was that as the software was designed to detect diffuse areas on the tooth surface using an algorithm based on a convex hull and therefore may be able to detect and quantify the diffuse areas of hypomineralization associated with fluorosis. The convex hull analysis software quantified the level of hypomineralization of the tooth surface image captured using the fluorescence imaging system. A number of stages were required in order to process the image (Figure 1d). The software was able to utilize the same masks of the object teeth created by a region of interest tool and employed by the existing technique. Prior to processing, the mask of the image was utilized in order to exclude any pixels outside of the tooth. The image reconstruction process was carried out in several stages. Firstly, the analysis software detected dark areas by reconstructing a “clean” image of the tooth surface and then subtracted the captured image. The reconstruction converted the image into a set of coordinates in the dimensions *x*, *y* and *brightness*. The convex hull of these points in these three dimensions was then calculated using the Quickhull algorithm written at the Geometry Center, University of Minnesota [18]. The convex hull was then converted back to an image using a simple software rendering algorithm. The result was an image of the tooth where dark areas were filled with an interpolation between surrounding areas. The map of fluorescence loss could then be thresholded to remove background noise, with all pixels below the threshold set to zero and all those above the threshold included in the map. In this study in order to include milder forms of fluorosis the threshold was set at a level of 5 (out of 255) pixels.

During analysis only the green channel was used and noise reduction was carried out by morphological opening before the reconstruction occurred. The development of the convex hull software and greater detail of the analysis processes are described in the literature [17]. Metrics were produced relating to the fraction of tooth area considered fluorosis (Area_ch_), the average fluorescence loss of areas considered fluorosis (ΔF_ch_) and the average fluorescence loss over the entire tooth surface (ΔQ_ch_).

Repeat fluorescence images captured for randomly selected subjects underwent a complete analysis procedure for both software analysis techniques. The same mask created from the repeat fluorescence images was utilized by both software analysis techniques to provide consistency with the main study data. The reproducibility data for the photographic assessments delivered a Kappa statistic of 0.80.

### Statistical analysis

The data for the photographic TF index scores from the epidemiological survey were entered into the Statistical Package for Social Sciences (SPSS 16.0) along with the metrics from the analysis of the fluorescence images using the existing technique and the convex hull software. For each subject, the higher of the two scores on the maxillary central incisors was used in the statistical analysis. Correlation coefficients between the photographic scores and the output from the software analyses were determined using for comparison with the QLF metrics (Area_blur_ ΔF_blur_ ΔQ_blur_ and Area_ch_ ΔF_ch_ ΔQ_ch_).

The data on cooking water fluoride content was converted into a categorical variable based upon concentration ranges separating the data into intervals. This is illustrated in Table 1. In order to assess the ability of either fluorescence image analysis technique to detect differences in fluoride exposure i.e. between each of the water intervals, a one-way ANOVA was conducted with a post-hoc correction for multiple comparisons. A non-parametric analysis using Mann-Whitney U Test would be employed if the assumptions for ANOVA were not upheld.

**Table 1:** Descriptive statistics for each cooking water interval for each of the metrics for fluorosis assessment

**Cooking water intervals (ppm)**		**Photographic**	**Convex hull software**			**Existing technique**		
		**TF index Score**	**Area**	Δ**F**	Δ**Q**	**Area**	Δ**F**	Δ**Q**
	N (%)							
<0.20	103 (18.6)	Mean 0.70	Mean 0.144	Mean 0.047	Mean 0.008	Mean 0.096	Mean 2.362	Mean 0.233
		SD 0.93	SD 0.105	SD 0.014	SD 0.007	SD 0.050	SD 0.482	SD 0.156
		Median 0	Median 0.099	Median 0.044	Median 0.004	Median 0.081	Median 2.255	Median 0.228
		Range 0-5	Range 0.025-0.464	Range 0.029-0.123	Range 0.001-0.033	Range 0.000-0.227	Range 1.294-2.983	Range 0.002-0.946
0.2 to 0.59	111 (20.1)	Mean 1.01	Mean 0.181	Mean 0.048	Mean 0.010	Mean 0.110	Mean 2.264	Mean 0.257
		SD 1.02	SD 0.130	SD 0.013	SD 0.010	SD 0.054	SD 0.455	SD 0.166
		Median 1	Median 0.153	Median 0.044	Median 0.007	Median 0.099	Median 2.183	Median 0.228
		Range 0-5	Range 0.023-0.555	Range 0.030-0.094	Range 0.007-0.046	Range 0.000-0.227	Range 1.356-2.364	Range 0.002-0.946
0.6 to 0.89	120 (21.7)	Mean 1.28	Mean 0.210	Mean 0.053	Mean 0.014	Mean 0.121	Mean 2.359	Mean 0.304
		SD 1.30	SD 0.151	SD 0.027	SD 0.020	SD 0.067	SD 0.542	SD 0.233
		Median 1	Median 0.163	Median 0.046	Median 0.007	Median 0.103	Median 2.283	Median 0.227
		Range 0-7	Range 0.024-0.635	Range 0.030-0.231	Range 0.001-0.146	Range 0.016-0.031	Range 1.477-4.414	Range 0.027-1.150
0.9 to 1.59	108 (19.5)	Mean 1.65	Mean 0.252	Mean 0.057	Mean 0.017	Mean 0.133	Mean 2.392	Mean 0.333
		SD 1.47	SD 0.162	SD 0.019	SD 0.016	SD 0.065	SD 0.494	SD 0.205
		Median 1	Median 0.206	Median 0.052	Median 0.010	Median 0.129	Median 2.331	Median 0.283
		Range 0-6	Range 0.039-0.678	Range 0.030-0.231	Range 0.001-0.080	Range 0.004-0.272	Range 1.324-3.873	Range 0.006-0.892
1.6+	111 (20.1)	Mean 2.30	Mean 0.299	Mean 0.062	Mean 0.022	Mean 0.155	Mean 2.592	Mean 0.424
		SD 1.90	SD 0.179	SD 0.026	SD 0.022	SD 0.075	SD 0.582	SD 0.289
		Median 2	Median 0.293	Median 0.056	Median 0.016	Median 0.150	Median 2.500	Median 0.359
		Range 0-7	Range 0.025-0.715	Range 0.030-0.203	Range 0.001-0.145	Range 0.001-0.340	Range 1.345-4.630	Range 0.002-1.399
Total	553 (100)							

## Results

Data for 560 subjects were available for analysis. After data cleaning 553 subjects were included in the analysis. Seven subjects were removed from the analysis owing to problems associated with processing the masks of the dentition. This occurred when there was either a missing mask (missing, fractured or restored incisor) or there was a large diastema between the central incisors. A decision was taken to exclude these subjects from the analysis rather than processing the image masks manually to ensure all images were analyzed using the same technique. Descriptive statistics for each of the assessment methods are described in Table 1. The subject distribution in each water interval was approximately equal. All of the outcomes demonstrated an increase in mean scores with increasing water fluoride content. The exception to this was the ΔF metric for the existing technique corresponding to the two water intervals with the lowest water fluoride content.

**Figure 2:**
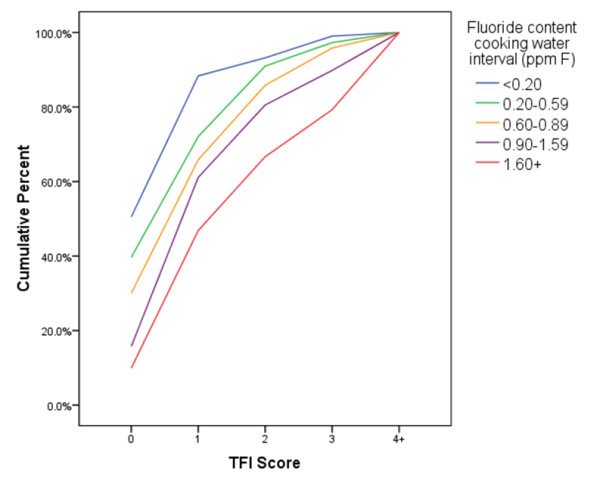
**The photographic score demonstrating separation of the intervals for cooking water fluoride content, suggestive of a dose response.** TF scores of 4 or higher have been grouped together as 4+.

**Figure 3:**
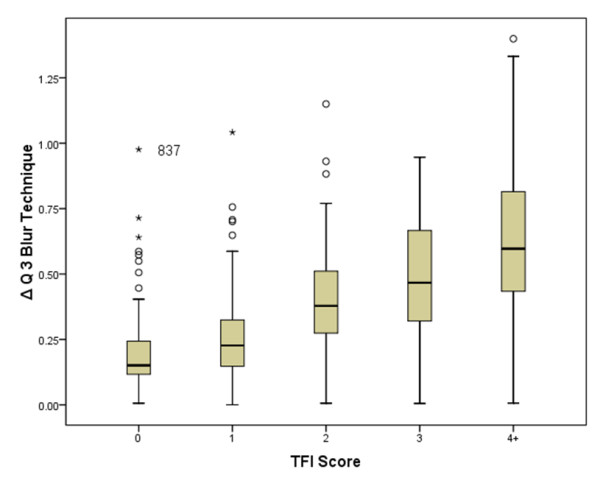
**Boxplot with error bars (SD) for ΔQblur.** Outliers (subject 837) highlighted.

**Figure 4:**
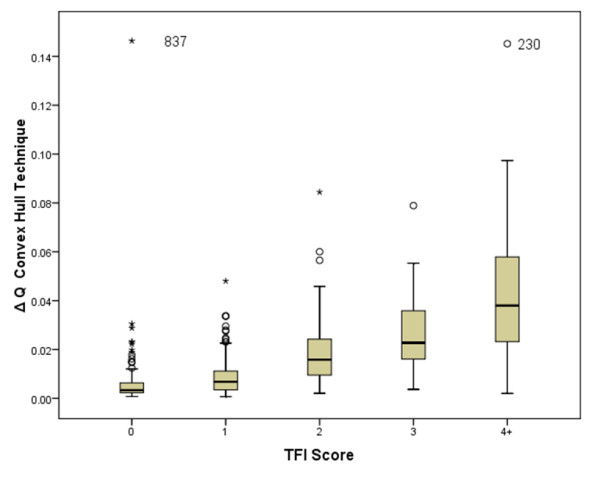
**Boxplot with error bars (SD) for ΔQch.** Outliers (subjects 837 and 230) highlighted.

The ability of the photographic scoring to detect differences in fluorosis severity at different exposures to fluoride is illustrated in Figure 2. Boxplots for the metric for ΔQ for both software analysis techniques demonstrated an increase in ΔQ as the TF index score increased (Figures 3 and 4). A one-way ANOVA between the photographic score and the fluorescence image analysis was performed with a Bonferroni correction for multiple comparisons. Analysis revealed the assumptions for ANOVA were not fully upheld; the assumption of homogeneity of variances was rejected (Levine’s test p < 0.05). In light of this a non-parametric analysis was performed using Mann-Whitney U Test with a simple Bonferroni correction for multiple comparisons. A summary of the ability of each technique to separate the water intervals is shown in Table 2. Overall, the convex hull software appeared to be almost as sensitive as the photographic score at discriminating between the water intervals when correcting for multiple pair-wise comparisons. The existing technique appeared to perform less well at lower water fluoride levels. All of the techniques performed less well for comparisons between water intervals 1 and 2 and water intervals 2 and 3.

**Table 2:** Pairwise comparisons for water fluoride intervals from cooking water and Photographic TF scores, “convex hull” and Existing method outcomes

**Photographic TF score Dependant variable water interval**			**Convex hull software Dependant variable water interval**			**Existing technique Dependant variable water interval**		
0	1	0.02	0	1	0.11	0	1	0.24
	2	<0.001*		2	0.004*		2	0.036
	3	<0.001*		3	<0.001*		3	<0.001*
	4	<0.001*		4	<0.001*		4	<0.001*
1	0	0.02	1	0	0.11	1	0	0.24
	2	0.11		2	0.18		2	0.34
	3	<0.001*		3	<0.001*		3	0.005*
	4	<0.001*		4	<0.001*		4	<0.001*
2	0	<0.001*	2	0	0.004*	2	0	0.036
	1	0.11		1	0.18		1	0.34
	3	0.049		3	0.016		3	0.076
	4	<0.001*		4	<0.001*		4	<0.001*
3	0	<0.001*	3	0	<0.001*	3	0	<0.001*
	1	<0.001*		1	<0.001*		1	0.005*
	2	0.049		2	0.016		2	0.076
	4	0.01		4	0.11		4	0.027
4	0	<0.001*	4	0	<0.001*	4	0	<0.001*
	1	<0.001*		1	<0.001*		1	<0.001*
	2	<0.001*		2		<0.001*		2	<0.001*
	3	0.01		3	0.11		3	0.076	

* *difference considered significant at the 0.005 level.

Water Intervals: 0 = < 0.2 ppm, 1 = 0.20-0.59 ppm, 2 = 0.60-0.89 ppm, 3 = 0.90-1.59 ppm, 4 = 1.6 + ppm.

The results of the correlations between the photographic scores and the fluorescence imaging output (Area, ΔF and ΔQ) are shown in Table 3. Both image analysis techniques demonstrated significant correlations with the photographic scores. Overall, the convex hull analysis software demonstrated a better association with the photographic scores than the existing technique for all outcome metrics. The metrics with the strongest correlations (Spearman’s rho) with the photographic score were Area_ch_ (0.66) and ΔQ_ch_ (0.65). The correlation for ΔQ_blur_ was still significant but was not as strongly correlated with the photographic scores (0.56). The correlation coefficient for the QLF metric ΔF for the existing technique was considered to be poor (0.30), although it should be stated the large sample size would have impacted on the statistical significance of the correlation coefficients.

**Table 3:** Correlation coefficients for each of the analysis software metrics compared to photographic TF score (n = 553)

**Software analysis metric**	**Spearman’s rho**	
	**Convex hull software**	**Existing technique**
Area	0.66**	0.59**
ΔF	0.54**	0.30**
ΔQ	0.65**	0.56**

** Correlation is significant at the 0.01 level (2-tailed).

An intraclass Correlation Coefficient (ICC) was obtained for each the fluorescence image analysis metrics. This data is illustrated in Table 4. The ICC for the convex hull software were all considered to be “very good”, the metric for ΔQ_ch_ delivering a value of 0.95. The values for the existing technique were slightly lower but still considered very good with a value of 0.85 obtained for the metric ΔQ_blur_.

**Table 4:** ICC for software analysis techniques (n = 44)

**Software analysis metric**	**Intra class correlation coefficients**	
	**Convex hull software**	**Existing technique**
Area	0.84**	0.80**
ΔF	0.96**	0.75**
ΔQ	0.95**	0.85**

** Correlation is significant at the 0.01 level (2-tailed).

## Discussion

The findings of this study support the potential use of fluorescence imaging to objectively quantify dental fluorosis. This is consistent with earlier work [15]. However, the correlation coefficients in the current study are lower than those obtained by Pretty et al [15]. This is probably due to the fact the population in the original study was a selected population based upon the presence of fluorosis in an area of optimal water fluoridation and presented with only milder forms of dental fluorosis. The population in the current study is larger and presents with a greater range of fluorosis severity and the increased presence of confounding factors. Nevertheless, the repeatability of both techniques is very good with the ICC for the existing technique being commensurate with the findings of Pretty et al and the convex hull software delivering even greater performance. This was achieved without employing techniques such as video repositioning and as such supports the claim that fluorescence image analysis can be robust in terms of the repeatability of measures [15].

There are certain considerations to be made regarding the population selected in this study. The population was selected according to the level of fluoride in their cooking water. Despite the fact the TF score obtained from the photographs was able to separate the different water fluoride content intervals (Figure 2) (suggestive of a dose response) it is clear this is not a true reflection of the fluoride exposure of the subjects. The risk to developing enamel fluorosis must include all forms of fluoride ingestion at the time of tooth development not only from cooking water but also drinking water, beverages, food and oral hygiene products [19-21]. It would be problematic to use total fluoride exposure to assess dose response in this population on this study and it should be accepted the use of cooking water fluoride content is not indicative when evaluating a dose response. However, this population was selected as lifetime residents and the likelihood the cooking water source had changed since birth was low. It had also been demonstrated that the current cooking water fluoride content was a strong measure when determining fluorosis risk [22].

Looking at the ranges of water fluoride content of the intervals (Table 1) intervals 0 and 1 could be seen to represent non-fluoridated populations with perhaps some background fluoride in water. Intervals 2 and 3 are commensurate with sub-optimal and optimally fluoridated populations with interval 4 representing fluoride levels above optimal levels. It would be desirable that any system would be able to discriminate between each of the intervals. However, it could be argued at the levels set in this study the difference between intervals 0, 1, 2 and 3 is minimal and the inability to discriminate between intervals 0 and 1 is not critical. However, a robust system should be able to discriminate between interval 4 and the remaining intervals.

Whilst the outcome of this study supports the development of fluorescence imaging as a technique for objectively quantifying enamel fluorosis, there remain several unresolved issues from the work of Pretty et al. Firstly there is still no acceptable gold standard to use. The use of the photographic score as the comparator remains inadequate as it depends upon a subjective assessment of fluorosis. The conventional digital photograph requires the camera to be position at an angle to the teeth (approximately 15° to the perpendicular plane) to reduce specula reflection, whereas the fluorescence imaging uses flat field illumination and polarizing filters enabling the images to be captured perpendicular to the teeth. This results in potential differences in the information that can be displayed between the images owing to foreshortening of the photographic image. Furthermore it is still not possible to relate the TF score from the photographs to the metrics obtained from either of the fluorescence analysis techniques. This is not a situation unique to the assessment of fluorosis, similar issues existed when fluorescence imaging was used for the assessment of carious lesions [23]. This would be true of any novel technique utilizing emerging technologies. Nevertheless, both fluorescence imaging techniques demonstrate an increase in ΔQ with increasing TF index score (Figures 3 and 4).

The decision to base the analysis on remote consensus scoring of standardized photographs was justifiable owing to the reduction of bias and examiner thresholding. The quality of the photographic images still enabled the detection of focal loss of surface enamel. Any issues associated with potential loss of validity were addressed by grouping the data for subjects with a TF score or 4 or higher. There is additional justification for this owing to the age range of subjects and the fact TF scores greater than 4 generally present as a result of post-eruptive changes to the fluorotic enamel.

The statistical analysis of the data is also compromised by the differences in the metric outputs. The correlation coefficients presented in this paper should not be regarded as a measurement of agreement as they are merely an indication of association between the different techniques. This is not only true of the comparison between the photographic scores and the fluorescence imaging but also between the two fluorescence imaging techniques. Despite similarities between the fluorescence imaging techniques, the methods by which the metrics are derived differ. The outputs whilst delivering the same outcome measures are presented using different scales.

**Figure 5:**
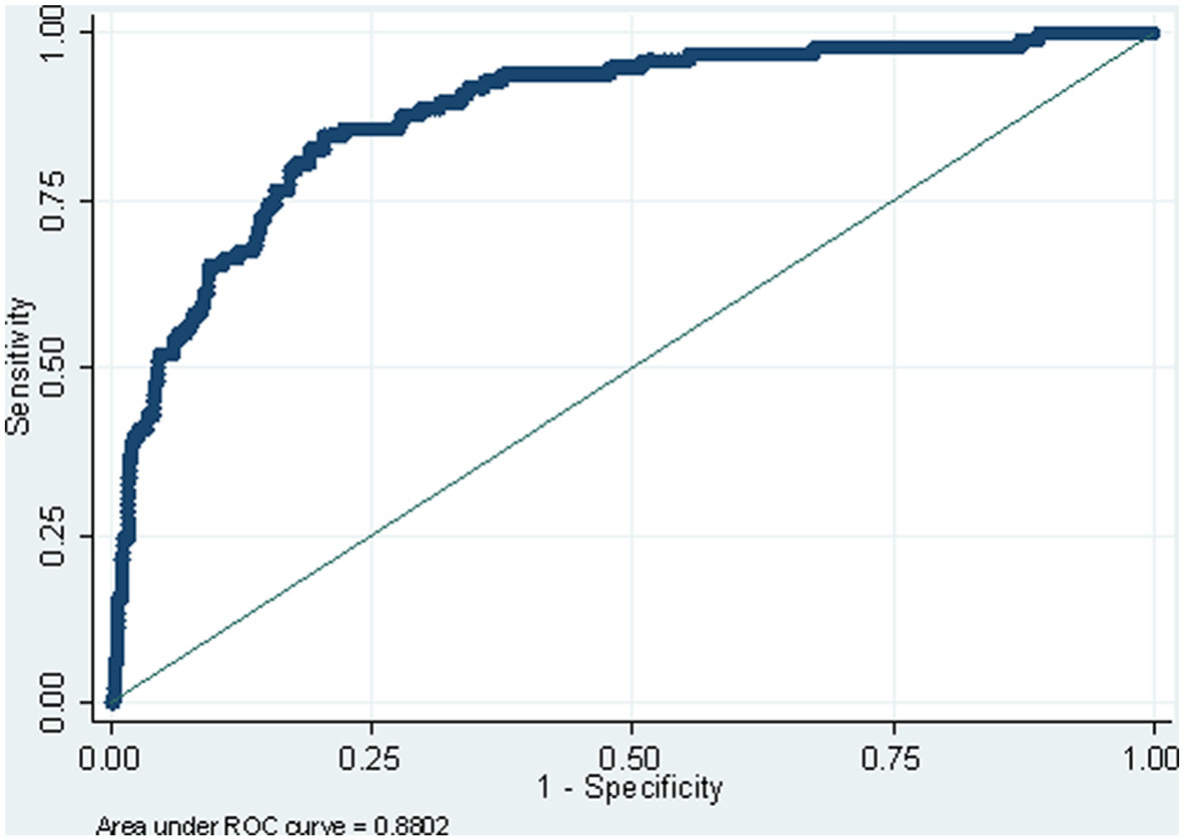
ROC curve for convex hull software.

**Figure 6:**
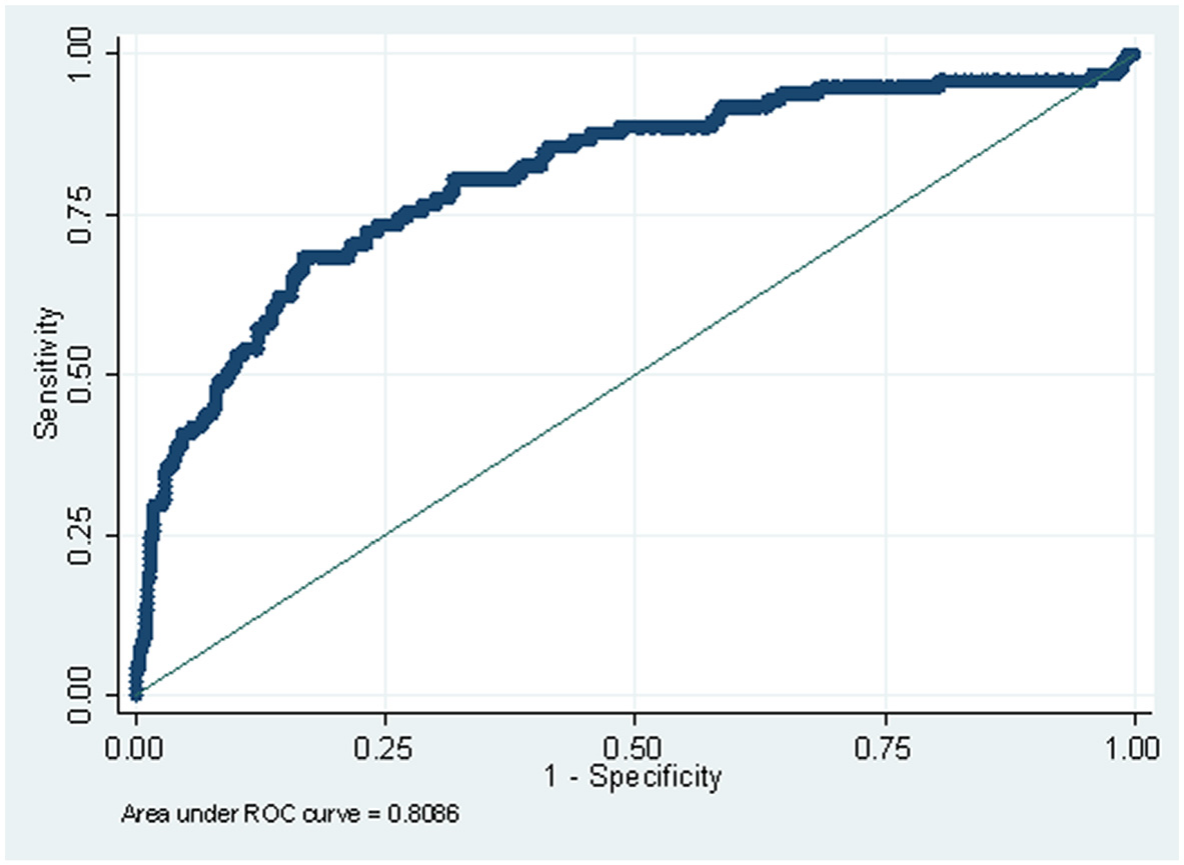
ROC curve for existing technique software.

All of the above factors contribute to difficulties in assessing the sensitivity and specificity of the fluorescent imaging technique and software analyses. In order to estimate the sensitivity and specificity of the fluorescent imaging system the data was exported to Stata (release 11, StataCorp, TX USA) and ROC curves produced using classification models for the QLF metric output ΔQ for each software analysis technique and a classifier boundary, or threshold, for fluorosis (TF score) of ≤2 and ≥3. These ROC curves are illustrated in Figures 5 and 6. The data would suggest (from this rudimentary assessment of sensitivity and specificity) the convex hull software demonstrated higher levels of sensitivity and specificity (sensitivity 80.61%; specificity 80.96%) when compared to the existing technique (sensitivity 68.37%; specificity 83.27%). The outcome was similar for estimating accuracy when comparing the area under the curve (AUC) for the convex hull and existing technique (0.8802 and 0.8086 respectively).

In order to reduce variance between the two fluorescence imaging techniques it was necessary to utilize the same masks of the teeth. The software in the original study required the operator to draw around the object teeth with a region interest tool. It is clear that repetition of this process could result in variance. Furthermore the original software required a reference area to be selected using the region of interest tool. This was overcome by using software written in Visual C# (2005 Express Edition, Microsoft, Inc., CA, USA) to process masks for all the object teeth from the fluorescence images. The software for the existing technique was augmented by the addition of an algorithm written in MATLAB that automatically selected a reference area from the triangulation of a point located on the gingival tissues with the masks of the maxillary central incisors (with an assumption of the location of the teeth). This algorithm worked well but was unable to process the analysis if there was either a missing mask (missing, fractured or restored incisor) or there was a large diastema between the central incisors. If this occurred the subjects and data were excluded from the analysis. This resulted in the exclusion of seven subjects.

**Figure 7:**
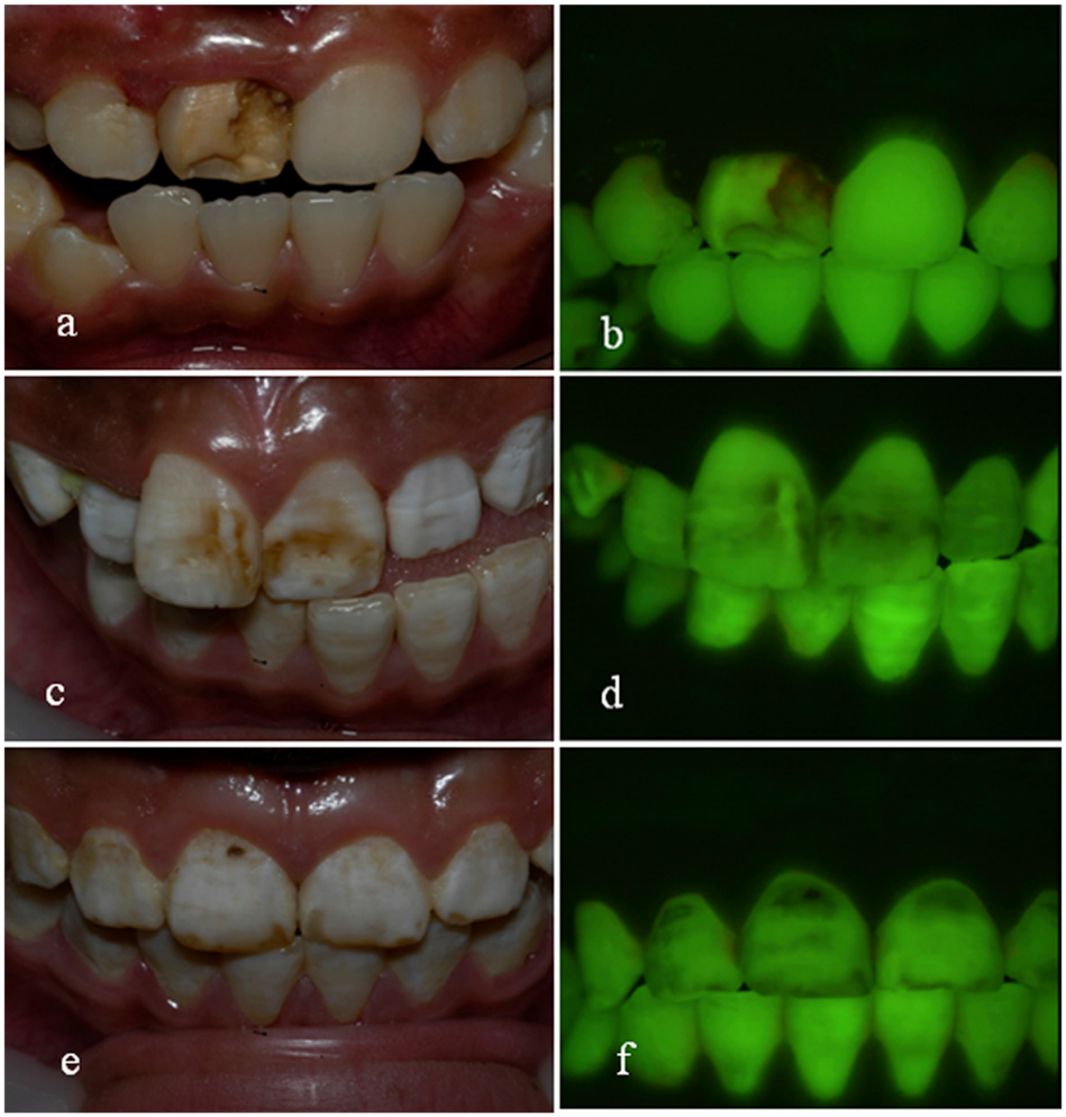
**Images of subjects with confounding factors for QLF. a**. clinical photograph subject 837 presenting with non-fluorotic hypomineralization and enamel loss on maxillary right central incisor. **b**. QLF image of subject 837. Note the pattern of fluorescence loss on the maxillary right central incisor typical of enamel loss with possible caries. The areas in red indicate presence of plaque stagnation. **c**. Clinical photograph of subject 230 presenting with confluent areas of fluorosis with pitting and staining. **d**. QLF image of subject 230. Areas of fluorosis with stain exhibit greater fluorescence loss. **e**. Clinical photograph subject 545 presenting with confluent fluorosis and enamel loss and possible caries. **f**. QLF image of subject 545. Note the loss of fluorescence in the areas of enamel loss.

The inability of the fluorescence imaging techniques to differentiate fluorosis from caries and other non-fluorotic developmental defects of enamel still exists. The subjects illustrated in Figure 7 demonstrate issues that can arise from this phenomenon. The images of subject 545 illustrate how the presence of caries and stain can impact upon the fluorescence image and subsequent analysis. The presence of plaque, stain, caries and other developmental defects of enamel such as demarcated enamel opacities are confounding factors in fluorosis assessment using fluorescence imaging [24]. It has been shown that demarcated opacities with similar clinical presentations can exhibit markedly different changes in fluorescence with some opacities demonstrating a loss of fluorescence whilst others demonstrating an increase in fluorescence signal.

Subject 837 (Figure 7) had suffered from a large developmental defect localized to the right maxillary central incisor with an aetiology non-fluorotic in nature. Both imaging techniques were unable to differentiate this from fluorosis and hence large values for Area, ΔF and ΔQ whereas the score allocated from the photograph for this subject was TF 0.

The images of subject 230 (Figure 7) illustrate fluorosis that has developed post eruptive stain. Whilst the existing technique was able to process this image the convex hull software was unable to differentiate the change in fluorescence relative to the surrounding unstained fluorosis and would have deemed the areas of discolouration as “heavy stain” and allocated a higher score for ΔF and ΔQ accordingly.

It is clear further work is needed if fluorescence imaging techniques are to be used for objectively quantifying fluorosis. It has been shown it can discriminate between populations with differing fluoride exposures. It is arguable which analysis technique is the more appropriate technique. The convex hull software would appear to be more sensitive than the existing technique at low fluoride exposures. This is likely to have been caused by the low threshold level set on this study. This was necessary to avoid excluding milder forms of fluorosis but would have included greater levels of noise in the analysis, affecting specificity. In fact the data suggests the ability of the convex hull software to discriminate between levels of fluorosis severity is comparable to the use of photographic scores. The existing technique appears to work well at higher severities of fluorosis. This is in contrast to the findings of Pretty et al who hypothesized that artifacts created by the existing technique may underestimate fluorosis. This may have been based on the findings from a population with lower exposures to fluoride and lower severities of fluorosis presentation. Overall both fluorescence image analysis techniques appear to be less sensitive than clinical judgment using an index when considering the whole range of presentations of fluorosis. Although in the case of the convex hull software this is marginal.

Although image capture is simple and reproducible it remains an additional step in study procedures. In addition, despite the fact the analysis is automated, there remains a considerable operator task in drawing the masks for image processing. At present it would appear the use of at least a photographic score using TF index and the application of diagnostic criteria cannot be dispensed with. The question arises as to what additional value can the use of fluorescence imaging provide over and above a clinical index? The answer may lie in the fact the longitudinal assessment of fluorosis is desirable and the variation in examiner scoring using a clinical index could be problematic when assessing prevalence and severity by clinical examination [9,10,25]. This can be avoided with the use of photographic scores, but the problem of subjectivity would remain.

Further software development is required particularly with respect to the production of the masks of the object teeth as this is the time dependant process that questions the viability of the application in a large epidemiological survey. Possible avenues to explore would be the production of automatic masks using edge detection software or more simply the use of preset polygons in Visual C# that can be adjusted to the shape of an object tooth rather than masks drawn freehand.

A possible interim solution could be to use a dual-camera system for image capture using two high resolution CCD cameras with an illumination and lens array that would permit one camera to capture a fluorescence image and a second to capture a polarized white light image (negating the need for camera repositioning to reduce specula reflection). Both sets of images would be of the same position relative to the teeth, same magnification and would both be amenable to longitudinal assessment through the use of video-repositioning software. Any white light image score using an index can remain blind and randomized and quantifiable metrics of fluorosis obtained from the corresponding fluorescence image.

## Conclusions

This study has shown that fluorescence imaging techniques can discriminate between populations with different fluoride exposures and a wide range of fluorosis severity. Both fluorescence image analysis techniques demonstrated very good levels of repeatability. The data support the early work in this field but further work is needed to develop the capturing system and software if it is to become a viable means of objectively quantifying fluorosis in large scale epidemiological surveys. At present there appears to be no means of avoiding the use of either the application of diagnostic criteria or the use of a clinical index in conjunction with fluorescence imaging for the objective quantification of fluorosis.

## Abbreviations

ANOVA: Analysis of variance; Area_blur_: Area of tooth considered fluorosis (existing/blur technique); Area_ch_: Fraction of tooth area considered fluorosis (convex hull technique); AUC: Area under the curve; CCD: Charge-coupled device; CI: Confidence interval; ICC: Intra-class Correlation Coefficient; ICOH: Intercountry Centre for Oral Health; LED: Light Emitting Diode; LR: Likelihood Ratio; RGB: Red, green, blue; QLF: Quantitative light induced fluorescence; TF: Thylstrup & Fejerskov Index; ΔF_blur_: Fraction fluorescence loss compared to sound enamel (existing/blur technique); ΔF_ch_: Average fluorescence loss of areas considered fluorosis (convex hull technique); ΔQ_blur_: Fraction fluorescence loss integrated by lesion area in mm^2^ (existing/blur technique); ΔQ_ch_: Average fluorescence loss over entire tooth surface (convex hull technique); ROC (curve): Receiver Operating Characteristic (curve).

## Competing interests

The authors declare that they have no competing interests.

## Authors’ contributions

MGM prepared the protocol, trained staff in the use of the QLF system, carried out remote TF scoring, performed the drawing of masks for QLF analysis, performed the analysis of data and wrote the manuscript. RPE inputted into the study design, trained staff in photographic methods, carried out remote TF scoring and inputted into the manuscript. PS & NK were local investigators responsible for photographic and QLF examinations. AT designed the QLF software and was involved in image analysis. MG provided statistical support and advice during data analysis and manuscript production. IAP inputted into study design and manuscript. All authors read and approved the final manuscript.

## Pre-publication history

The pre-publication history for this paper can be accessed here:

http://www.biomedcentral.com/1472-6831/12/33/prepub
